# Comparative Laser Spectroscopy Diagnostics for Ancient Metallic Artefacts Exposed to Environmental Pollution

**DOI:** 10.3390/s100504926

**Published:** 2010-05-14

**Authors:** Łukasz Ciupiński, Elżbieta Fortuna-Zaleśna, Halina Garbacz, Andrzej Koss, Krzysztof J. Kurzydłowski, Jan Marczak, Janusz Mróz, Tomasz Onyszczuk, Antoni Rycyk, Antoni Sarzyński, Wojciech Skrzeczanowski, Marek Strzelec, Anna Zatorska, Grażyna Z. Żukowska

**Affiliations:** 1 Faculty of Materials Science and Engineering, Warsaw University of Technology, 141 Wołoska Street, Warsaw 02-507, Poland; E-Mails: lciupinski@inmat.pw.edu.pl (Ł.C.); elaf@inmat.pw.edu.pl (E.F.-Z.); hgarbacz@inmat.pw.edu.pl (H.G.); kjk@inmat.pw.edu.pl (K.J.K.); tonyszczuk@gmail.com (T.O.); 2 Inter-Academy Institute for Conservation and Restoration of Works of Art, Academy of Fine Arts in Warsaw, Wybrzeże Kościuszkowskie 37, 00-379 Warsaw; Poland; E-Mails: kossa@asp.waw.pl (A.K.); janusz.mroz@op.pl (J.M.); aniasz11@wp.pl (A.Z.); 3 Institute of Optoelectronics, Military University of Technology, 2 Gen. Sylwestra Kaliskiego Street, 00-908 Warsaw, Poland; E-Mails: jmarczak@wat.edu.pl (J.M.); arycyk@wat.edu.pl (A.R.); asarzynski@wat.edu.pl (A.S.); wskrzeczanowski@wat.edu.pl (W.S.); 4 Faculty of Chemistry, Warsaw University of Technology, 3 Noakowskiego Street, 00-664 Warsaw, Poland; E-Mail: zosia@ch.pw.edu.pl

**Keywords:** metal artworks, environmental pollution, laser spectroscopy, artwork diagnostics

## Abstract

Metal artworks are subjected to corrosion and oxidation processes due to reactive agents present in the air, water and in the ground that these objects have been in contact with for hundreds of years. This is the case for archaeological metals that are recovered from excavation sites, as well as artefacts exposed to polluted air. Stabilization of the conservation state of these objects needs precise diagnostics of the accrued surface layers and identification of original, historical materials before further protective treatments, including safe laser cleaning of unwanted layers. This paper presents analyses of the chemical composition and stratigraphy of corrosion products with the use of laser induced breakdown spectroscopy (LIBS) and Raman spectroscopy. The discussion of the results is supported by material studies (SEM-EDS, XRF, ion-analyses). The tests were performed on several samples taken from original objects, including copper roofing from Wilanów Palace in Warsaw and Karol Poznański Palace in ŁódŸ, bronze decorative figures from the Wilanów Palace gardens, and four archaeological examples of old jewellery (different copper alloys). Work has been performed as a part of the MATLAS project in the frames of EEA and Norway Grants (www.matlas.eu) and the results enable the comparison of the methodology and to elaborate the joint diagnostic procedures of the three project partner independent laboratories.

## 1. Introduction

As far as quantity is concerned, metal monuments and works of art are among the largest material groups for cultural heritage objects. Copper and its alloys play an important role as an original core material amongst a very wide assortment of various metals and their alloys used by artists in the past. Apart from accidental damage, long-term use or deposited dirt, the most typical type of deterioration seen in metal artifacts is the result of chemical changes due to the reactivity of chemicals from many sources in the environment. However, alteration and corrosion processes on archaeological findings are quite different with respect to metal artefacts exposed to atmospheric aggression. The process that causes the formation of corrosion products on archaeological copper alloys is usually complex and varies depending on the burial milieu and material composition [1–3]. Such objects show a variety of superficial formations that form a brittle encrustation surface layer. In most cases green calcareous accretions with embedded silicates completely overlay the oxidation patina (typically cuprite) [4]. Weathered metal artwork surfaces suffer substantially from the impact of atmospheric pollutants. Permanent contact with atmospheric pollution, temperature and climate changes cause serious soiling, acid metal corrosion as well as lots of mechanical damage [5,6]. Metal objects exhibited in the open air inside built-up areas suffer mainly from soot and dust that has been emitted to the atmosphere or has risen from the earth surface as well as from motor exhaust gases and substances generated by modern industry. The surfaces of many copper artworks are gilded using various techniques like fire, leaf or electrochemical gilding [7]. In many cases, the gold layer is damaged because of corrosion or other aging processes such as the development of cracks (craquelure) in the ground layer of leaf gildings [8].

Historical copper and copper alloys have been studied recently and in the past on a regular basis, using various techniques with emphasis on the surface alterations they have suffered in the course of time [9–12]. As a part of a broader project [13], aimed at investigating the different characteristics of metal artworks before and after laser cleaning experiments, this paper presents the corrosion studies results, obtained by means of laser Raman and LIBS spectroscopy, which have already proven to be valuable tools for the evaluation of different works of art [14–17]. The complex nature of the formed corrosion products and limited versatility of any diagnostic method requires a combination of several surface analytical techniques for the complete characterization of encrustation layers. In addition to Raman and LIBS spectroscopy, the objects were examined using optical microscopy (micro-sections), micro-chemical analyses (ion composition) and energy dispersive X-ray spectroscopy (SEM-EDS).

## 2. Artworks and Diagnostic Methods

### 2.1. Historical Objects

Metal artefacts to be investigated in this work were selected from several collections that have been made available for diagnostics and laser cleaning tests within the framework of the MATLAS project.

**Figure 1.**
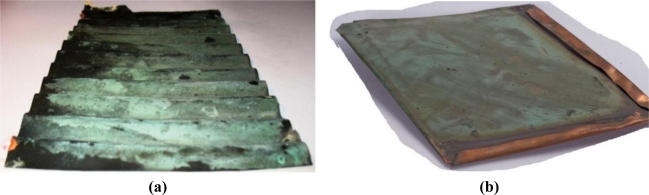
Old copper roofing samples: (a) from Wilanów Palace in Warsaw; (b) from the dome of Karol Poznański Palace in ŁódŸ, Poland.

A set of corroded copper coupons from old roofing (Figure 1) are excellent examples of weathered metal artwork surfaces, which have suffered tremendously from the impact of atmospheric pollutants. The samples originated from the Wilanów Palace in Warsaw (the turn of the 19th century) and the Karol Poznański Palace in ŁódŸ (beginning of the 20th century), both are copper roof sheets that have been recently replaced. These objects exemplify excellent research potential, both by the volume and the area of material, and due to the different state of conservation for each of the opposed sides.

**Figure 2.**
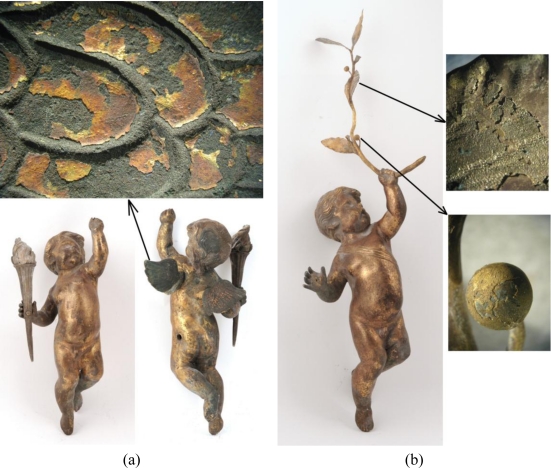
Pair of gilded bronze putti from the Wilanów Palace garden façade: (a) putto with torch, above—microscopic photograph of damage to the putto’s wing; (b) putto with laurel, left side—microscopic photographs of damage to the fruit and leaf.

Figure 2 shows a further two objects, a pair of gilded bronze putti, dated to the end of the 17th century and attributed by annotations to the Rome studio of the Dutch sculptor Disqenue. Their function as decorative elements of the Wilanów Palace garden façade has caused serious soil, local corrosion of the alloy under the gilding as well as numerous mechanical damages. Both figures have been included in the full conservation program with the use of both traditional and laser cleaning technology.

**Figure 3.**
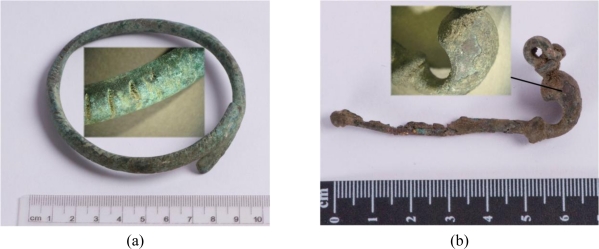

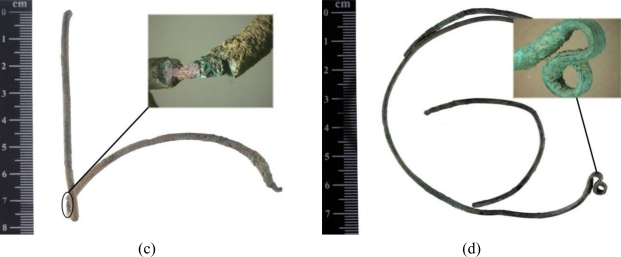
Archaeological artworks made of copper alloys: (a) bracelet; (b) fibula, property of the Jasna Góra Monastery, Częstochowa; (c-d) bows, property of the Wilanów Palace Museum in Warsaw.

The third group—jewellery—is represented by four archaeological findings, currently belonging to the collections of the Wilanów Palace Museum and the Pauline Fathers’ Jasna Góra Monastery (Figure 3). Two bows from the Wilanów collection were found at a medieval graveyard near the Orangery Palace, dated to the 12th/13th century. The ancient fibula and bracelet of unknown provenance are oblations given to Jasna Góra Monastery in Częstochowa. As in the case of other reported archaeological bronze jewellery and coins [4], these samples present various alterations, typically appearing as superficial formations and encrustations due to both products of metal corrosion and exogenous materials.

### 2.1. Experimental Procedures

Prior to analyses, sections of each object were removed, embedded in a Meliodent epoxy resin and polished. The resulting micro-sections were observed in a reflected light using a Zetopan microscope and registered with a SMZ-2T stereo-microscope and Nikon COOLPIX MDC photographic package. Apart from the microscopic observations, preliminary identification of corrosion products composition using acidic and alkaline treatments and ion analyses was performed.

Raman spectra of corrosion products were registered using a dispersive Nicolet Almega Raman spectrometer, equipped with two laser illumination sources (532 nm and 780 nm), Olympus BX research-grade confocal microscope (magnification 50x, long FD) and high precision motorized x-y stage. Nominal laser power of 25 mW (532 nm) and 35 mW (780 nm) was usually reduced (to around 30%) due to the small diameter of the focused laser spot (∼2 μm) and sensitivity of the samples. Exposition time was 15–60 s in each of the two averaged scans of the sampled areas. Species identification followed application of the Thermo Scientific OMNIC Specta software.

**Figure 4.**
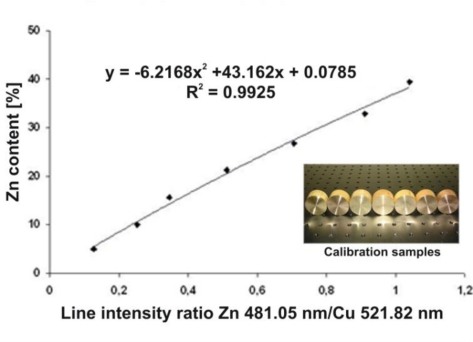
LIBS calibration characteristic for Zn-Cu alloys.

The essential part of the LIBS measurement system is the ESA 4000 *echelle* type spectrometer with Kodak K1001 ICCD camera. The instrument ensured spectral resolution of λ/Δλ = 15,000 in the measurement range of 200–785 nm. The spectrometer was equipped with two diffraction elements—a prism as a diffraction order sorter and an echelle type diffraction grating used as the main dispersive element, which gave very high radiation intensity in diffraction orders and a very good spectral resolution. Plasma was generated by the Nd:YAG Brio model laser (BigSky/Quantel) operating at the fourth harmonic 266 nm (11.2 mJ, 4 ns). Laser radiation was focused on the objects using a 100 mm quartz lens. The typical lens-to-target distance was 97 mm in order to avoid laser breakdown in the air (laser spark) and to ensure stable, repeatable energy density at the laser spot. Recorded spectra were integrated in 10 μs time windows for a fixed delay of 1,000 ns with respect to the plasma generating laser pulse, in order to increase the line to continuum radiation ratio (line radiation dominated continuum with this delay). Each stratigraphy result was derived from one shot for the Wilanow and ŁódŸ palace roofings and the putti figure, while for archaeological artefacts each result was obtained from 20 accumulations, due to the large encrustations covering the analysed objects. Quantitative measurements of the object substrate composition were performed on the basis of calibration characteristics [18], registered for known standard samples of metal alloys using similar experimental conditions and compositions of the same main metal components. An example of the calibration characteristic for Zn-Cu alloys is given in Figure 4.

Comparative examinations of the superficial structures and their chemical composition were performed using two SEM-EDS systems:

- JEOL JSM-6380LA. The EDS, preliminary and qualitative only measurements were performed at 20 kV.
- HITACHI S-3500N. The investigations included observations in SE and BSE modes and point analyses of the chemical composition by EDS. The EDS measurements were performed at 15 kV and 30 kV.

For XRD examinations the deposit was removed from the surface of the object and further analysed in powder form. The phase composition of the deposit removed from the surface of the copper sheet was determined with a Philips 1830 X-ray diffractometer (XRD) with X-Pert goniometer using CuK_α_ radiation. The measurement was performed in a Θ/2Θ geometry, the 2Θ range was 20–100°, step 0.05° and sampling time of 3 s per step.

## 3. Experimental Results and Discussion

### 3.1. Corroded Copper Sheets

**Figure 5.**
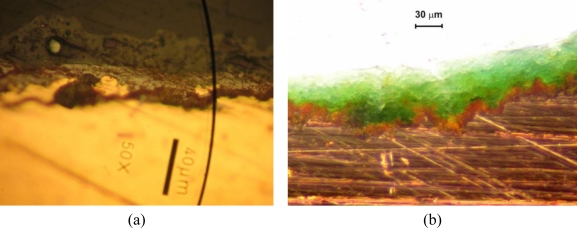
Polished cross-sections of the encrustation on copper sheets: (a) from Wilanów Palace in Warsaw (digital camera Canon Power Shot A 640); (b) from Karol Poznański Palace in ŁódŸ (stereo-microscope SMZ-2T).

**Figure 6.**
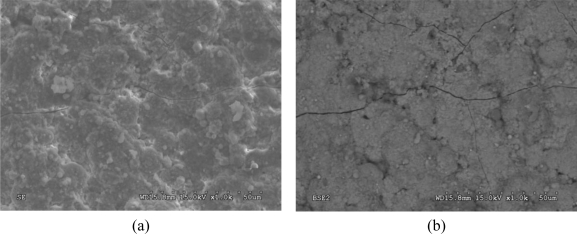
Sample SEM images of the deposit surface topography and composition of the sample from Wilanów Palace: (a) SE topographical mode; (b) BSE mode showing the chemical composition (dark areas contain lighter elements).

The surface appearance and cross-sections of the copper roofing samples were examined using SEM and optical microscopy. Corrosion products and encrustation are similar to a large extent on both objects (Figure 5). Figure 6 shows SEM photographs of the surface of copper sheets from Wilanów Palace in Warsaw. The chemical composition of the top part of the deposit is presented in Table 1. The main components of the layer are copper, oxygen and sulphur. Small amounts of aluminium, silicon, chlorine, iron and phosphorus were also detected.

The SEM images of the encrustation layer cross-section revealed embedded particles in its upper sub-layer, with dimensions of up to 30 μm. This part of the deposit was brittle. The EDS analysis revealed the presence of copper and oxygen in the bottom part of the layer. The upper part of the deposit is rich in copper, oxygen and sulphur. The chemical composition of the embedded particles indicates alumino-silicates /silicates (quartz).

**Figure 7.**
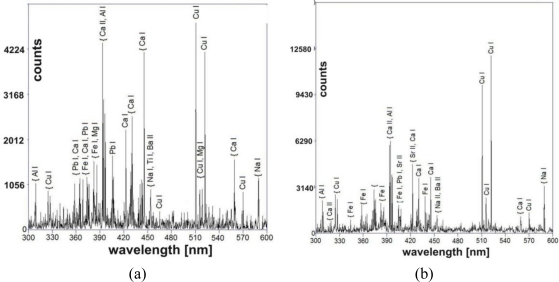
LIBS spectra of cooper sheets with deposit registered during the first laser shot on the black part of samples: (a) sample from the Wilanów Palace roofing; (b) sample from the Palace roofing in ŁódŸ.

The surface EDS measurements presented so far refer to the green part of the deposit with a small amount of calcium. Figure 7 shows the results of LIBS analysis on copper sheets, taken during the first laser shot. As both measurements were performed in different periods of time and experimental conditions, they can’t be compared quantitatively, however both spectra reveal almost the same elementary composition of the deposits. In comparison with EDS measurements (Table 1), a substantial increase in the concentration of Ca can be observed. The presence of calcium is characteristic for black atmospheric deposits on surfaces that are sheltered from rain washing.

**Figure 8.**
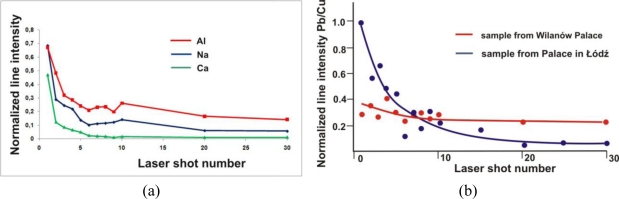
Results of in-depth LIBS analyses of elements in the copper roofing deposit: (a) Al, Na and Ca from the Palace in ŁódŸ sample; (b) Pb from both samples.

Figure 8 shows in-depth distribution of elements in the deposit measured using the LIBS method with a sequence of laser pulses. The line intensities of Na I 589.59, Al I 394.40, Ca I 445.48, Fe I 371.99 nm and Pb I 405.78 nm were normalized to the intensity of the copper line Cu I 510.553 nm, which was the substrate material. As a measure of the laser sampling depth the number of laser shots at the same place was assumed. As it has been determined during earlier metal calibration experiments and tests of ablation efficiency (SEM analysis), a layer of 5 to 10 μm is ablated by one, small energy laser pulse depending on the material structure. As can be seen in Figure 8(a), the calcium was concentrated mainly in the upper part of the encrustation, inside a relatively thin layer with a maximum thickness of 100 μm, while the removal of Al and Na needed dozens of laser shots. What is interesting is the relative change in lead content (see Figure 8(b)), which may indicate a higher concentration of air pollutants containing lead at the location of the Palace in ŁódŸ than at the location of the palace in Wilanów. Wilanów Palace in Warsaw is situated between Wilanów Park and Wilanów Gardens, and is quite a distance from the hustle and bustle of the metropolis. Karol Poznański Palace in ŁódŸ on the other hand is situated at the intersection of busy streets and higher lead concentrations are usually assumed to originate from vehicle exhaust emissions.

**Figure 9.**
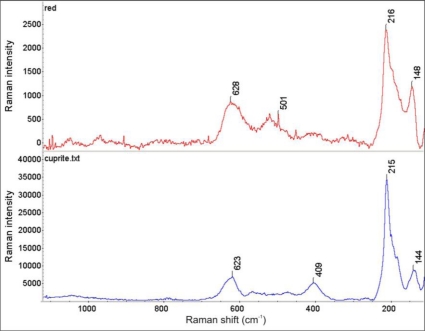
Comparison of the Raman spectrum of cuprite Cu_2_O (lower plot) with the Raman spectrum of the red corrosion layer shown in Figure 5 (upper plot).

**Figure 10.**
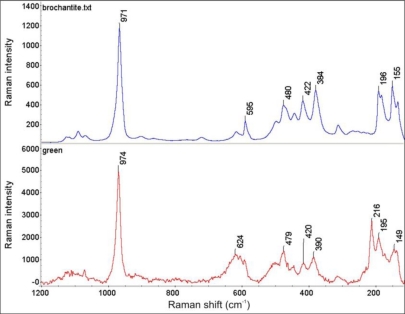
Comparison of the Raman spectrum of brochantite Cu_4_SO_4_ (OH)_6_ (upper plot) with the Raman spectrum from the green corrosion layer shown in Figure 5 (lower plot).

Raman spectra of clearly separated red and green layers from the Wilanów Palace roofing (Figure 5) are shown in Figures 9 and 10, indicating, respectively, the presence of cuprite Cu_2_O and brochantite Cu_4_SO_4_ (OH)_6_. This matches the two main phases that copper goes through during natural weathering [20]. Cuprite is the initial corrosion product and it is always the patina layer in contact with the copper. Growth laws that describe the patina formation indicate the decrease in the corrosion rate with an increase in exposure time due to the protective nature of the cuprite layer. The green patinas are typically characterized by an outer layer of brochantite, which forms as individual crystals on the surface of the cuprite layer, probably by a precipitation reaction from an aqueous surface layer on the cuprite layer [21].

**Figure 11.**
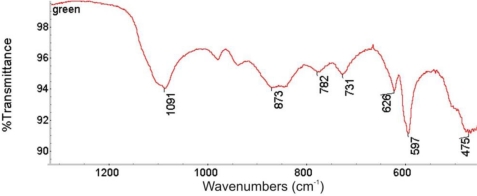
FTIR spectrum of the green layer shown in Figure 5.

Identification of brochantite has been confirmed by the FTIR spectrum (Figure 11), registered using a Perkin Elmer System 2000 (resolution 2 cm^−1^, 32 scans averaging).

The analysis of the XRD patterns obtained for the deposit removed from the surface also allowed for the identification of cuprite and brochantite as major corrosion phases [22], however traces of antlerite Cu_3_(SO_4_)(OH)_4_, hydrogen sulfide H_2_S, paramelaconite Cu_4_O_3_ and copper carbonate have also been detected.

### 3.2. The Two Putti Figures

Studies of the encrustation structure and composition were performed on both putti figures in a number of places, however due to the limited number of pages in this paper only the most informative and representative results are presented here.

**Figure 12.**
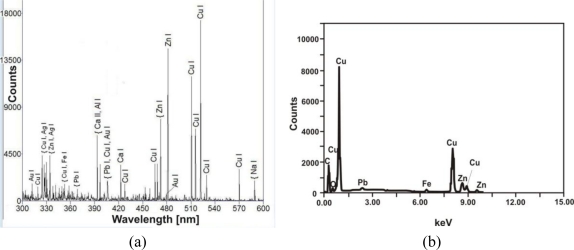
Putto with laurel substrate alloy: (a) LIBS spectrum; (b) EDS spectrum.

The putti substrate is an alloy of copper and zinc (11–20 wt%) with small admixtures of lead (0.2–1.4 wt%). Figure 12 shows representative examples of LIBS and EDS spectra. LIBS measurements gave two important conservation indications. The first concerns the structure of the putti figures-different parts were made of slightly different copper alloys, which can suggest a secondary origin for some of the decorative elements. The second is probably connected with previous conservation procedures for the putto with the laurel. As can be seen in Figure 13, the presence of the barium, strontium and titanium lines in the LIBS spectrum indicate overpainting of the laurel branch with a mixture of barium and strontium yellow with titanium white (gilding reproduction).

**Figure 14.**
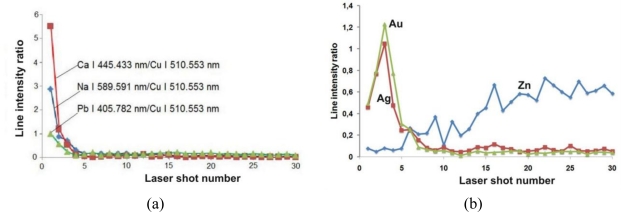
Results of in-depth LIBS analysis at of the laurel putto leaf: (a) distribution of “environmental elements” (deposit); (b) distribution of gilding elements and Zn.

Stratigraphic LIBS measurements have shown that all putti elements are covered with an encrustation that contains mainly calcium, sodium, aluminium and lead. Lead is also one of the original alloy elements, but its higher concentration in the superficial layers indicates the influence of atmospheric pollution. In-depth changes of individual elements on the surface are shown in Figure 14. All line intensities are normalized to the intensity of copper line Cu I 510.553 nm, which is the main component of the substrate material. Again, the measure of the laser sampling depth is the number of laser shots at the same measurement spot.

Stratigraphic changes in the relative compositions of Na, Ca and Pb at the painted layer of the laurel leaf are shown in Figure 14(a). A similar distribution is characteristic for all the putto figure elements (body, wings, laurel) and also applies to the Al and Mg content. All these elements have accumulated inside a very thin superficial layer (usually up to 50 μm) and influence the stratigraphic distribution of the original materials of the outer sculpture area (e.g., gilding). This can be seen in Figure 14(b), which presents similar distributions of Au, Ag and Zn. The initial increase of the relative Au and Ag concentrations is caused by gradual removal of the outermost deposit (Figure 8). The following decrease in the plot lines should be attributed to the relatively small thickness of the layers (10–20 μm). The in-depth resolution of LIBS stratigraphy is too low to separate and define the sequence of the Au and Ag layer positions. Traces of the relative Zn content shows in turn penetration of the laser beam through the whole encrustation after around 20 shots (∼200 μm).

**Figure 15.**
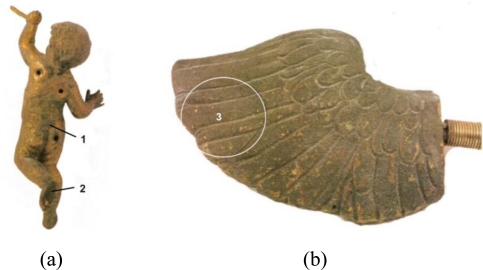
Indication of the measurement points: (a) body of putto with laurel (without wings)—back side; (b) left wing of putto with torch.

**Figure 16.**
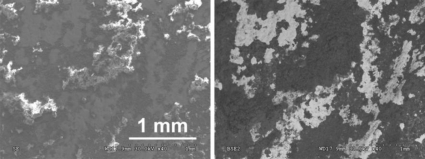
SEM images of the surface of the wing of the putto with the laurel (Figure 15) in SE mode (left) and BSE mode(right).

**Figure 17.**
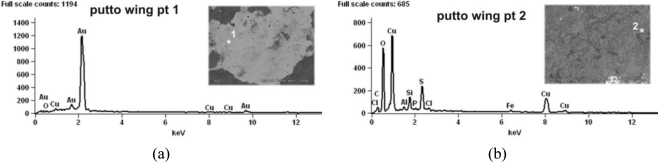
EDS spectrograms of the chemical composition at the points indicated by the numbers (small SEM photographs): (a) gilding; (b) corrosion.

The surface composition of the samples was examined using a scanning electron microscope HITACHI S-3500N equipped with EDS. Figure 15 presents the sampling points. Rresults of the elementary analyses are summarized in Table 2. Observation of the surface of the left wing (putto with laurel—area 3 in Figure 15) revealed that most of its surface is covered by corrosion products. This is shown in the images registered by the secondary electrons (SE) mode as accretions (uplifts), while the surrounding flat areas correspond with the preserved gilding (Figure 16 photo on the left). Dark places, registered by the backscattered electrons (BSE) mode, correspond to corrosion products (Figure 16 photo on the right). The EDS spectra revealed the presence of products of copper, oxygen and sulphur, the latter present at a level of several weight percent, which indicates copper sulphates. The particles embedded into the deposit were also studied (Figure 17 (a)). A chemical analysis of the particles indicates the presence of alumino-silicates and silicates (quartz). Furthermore, the presence of magnesium (0.6 wt%), potassium (0.2–1 wt%), phosphor (0.2–0.9 wt%), and carbon have been locally detected. Studies of places where the gilding was preserved (Figure 16) revealed numerous surface discontinuities and damage.

**Figure 18.**
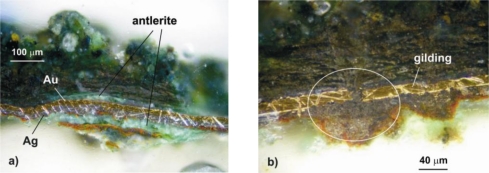
Cross sections of micro-samples from point 1 (Figure 15a): (a) presence of Ag and Au layers; (b) defect in the gilding layer accomplished by a large growth of corrosion products.

More informative, two-dimensional structures and the composition of the samples were studied using their polished microsections that had been embedded in the acrylic resin Meliodent. This has allowed for the determination of the stratiform image of the encrustation and gilding, the thickness of its components and to perform further Raman analyses. As shown in Figure 18, a section from a layer of the delaminated gilding (point 1 in Figure 15(a)) revealed two metallic coatings—silver and gold. Corrosion products were observed below and above the gilding, growing substantially in places of flows (Figure 18(b)). Classical micro-chemical analysis (water smears, ionic reactions) detected ions of Cu^2+^, S^2−^, SiO_3_^2−^, Au^3+^ and Ag^1+^, particularly in the black part of the encrustation (point 2 in Figure 15a and area 3 in Figure 15(b)). This can suggest the presence of black copper sulfide. These results conform with the data in Table 2 (point 2).

**Figure 19.**
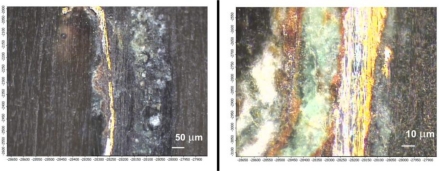
Section of sample from point 1 in Figure 15. Photographs registered using a Raman system microscope (section presented in Figure 18).

**Figure 20.**
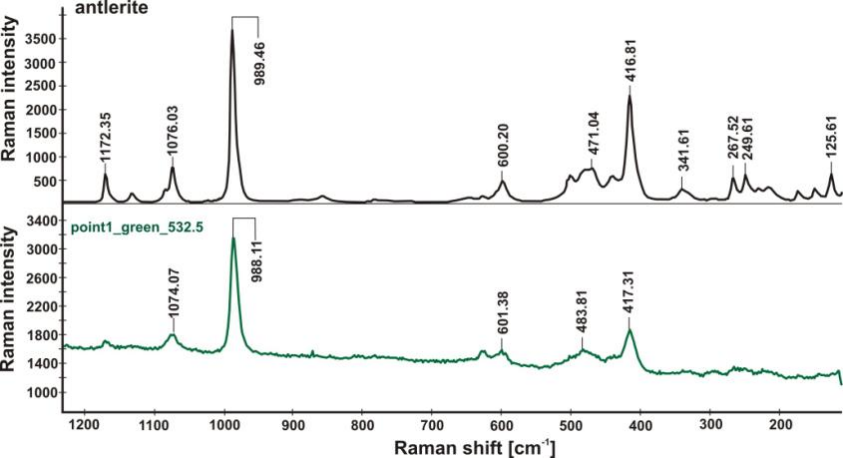
Comparison of the Raman spectrum of antlerite Cu_3_SO_4_(OH)_4_ (upper plot) with the Raman spectrum of green corrosion layer shown in Figure 18 (lower plot).

**Figure 21.**
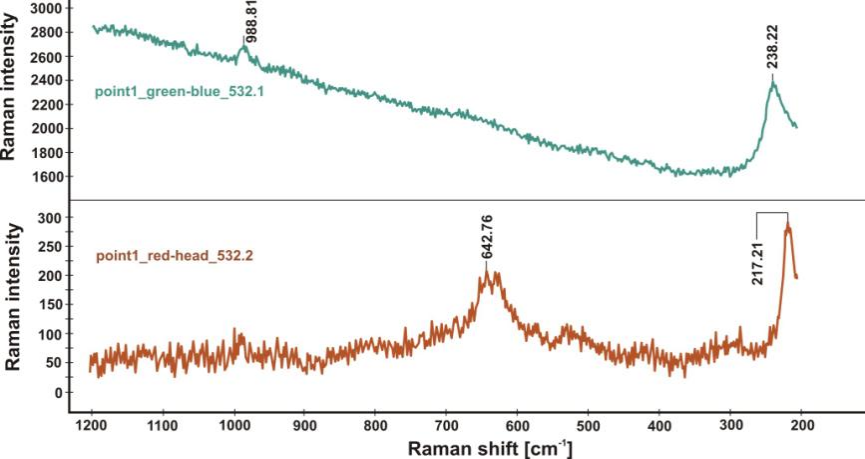
Raman spectra from the blue-green layer (upper plot) and red-brown layer (lower plot) shown in Figure 18, indicating the presence of antlerite (988.81 cm^−1^), cuprite (217.21 cm^−1^) and probably silver oxide Ag_2_O (238.22 cm^−1^) [19].

**Figure 22.**
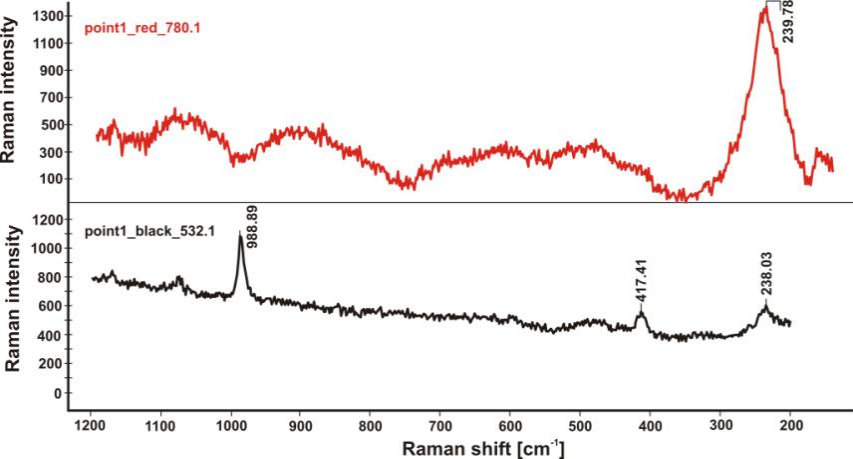
Raman spectra from the red layer (upper plot) and black layer (lower plot) shown in Figure 18, indicating the presence of antlerite (988.81 cm^−1^, 417.41 cm^−1^), and probably silver oxide Ag_2_O (239.78 cm^−1^, 238.03 cm^−1^).

Application of Raman spectrometry allowed for the further identification of several corrosion compounds. Raman spectra, registered at section of point 1 in Figure 15a (magnification shown in Figure 19) are presented in Figures 20–22. Figure 20 presents the Raman spectrum of green copper salt, which has been identified as antlerite Cu_3_SO_4_(OH)_4_ (Figure 20: upper plot), and can be found below and above the metallic layers in Figure 18a. Cuprite Cu_2_O has been identified as the red compound in Figure 18, and the grey salts there are probably silver compounds (silver sulfates), which were not identified in Raman spectra, but ions of S^−2^ and Ag^+1^ have been found during microchemical analysis. Bands near 235 cm^−1^ probably indicate the presence of Ag_2_O.

### 3.3. Archaeological Objects

The archaeological objects (Figure 3) presented various alterations, typically appearing as superficial formations and encrustations due to both the products of metal corrosion and exogenous materials. Prior to further, preliminary analysis of the surface deposits and corrosion, LIBS measurements were performed to determine the composition of the original alloys. All line intensities were normalized to the intensity of copper line Cu I 515.323 nm, which was narrower than line Cu I 510.553 used in the case of copper sheets and putti figures. The component line wavelengths were: Fe I 385.991 nm, Zn I 481.053 nm, Sn I 286.332 nm, Pb I 405.781 nm.

Presentation of the results of the corrosion studies are divided into two sections, according to the two, probably comparable origins of both pairs of archaeological findings. It should be emphasized that this part of the research is still in progress, and the results presented here should not be treated as a complete characterization of these objects. In particular it relates to the Raman spectra, which are sometimes too complex to perform the final identification of the components. Similar problems with the assessment of Raman spectra on archaeological corrosion have been reported in literature [23]. Sometimes it was necessary to set up a library of reference spectra [24].

#### 3.3.1. Bracelet and Fibula from Jasna Góra Monastery, Częstochowa

**Figure 23.**
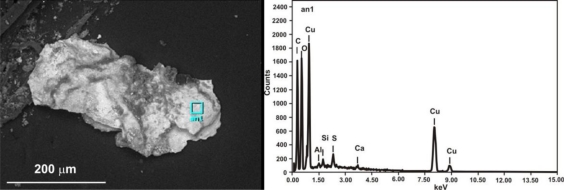
Bracelet micro-sample SEM photograph (on the left) and EDS spectrum (on the right).

**Figure 24.**
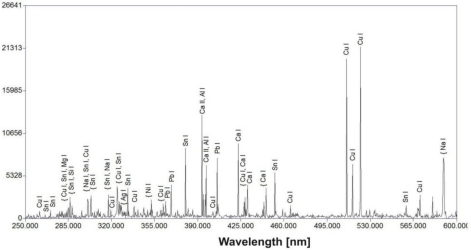
Encrustation LIBS spectrum of the bracelet registered during the first accumulation of twenty laser shots.

**Figure 25.**
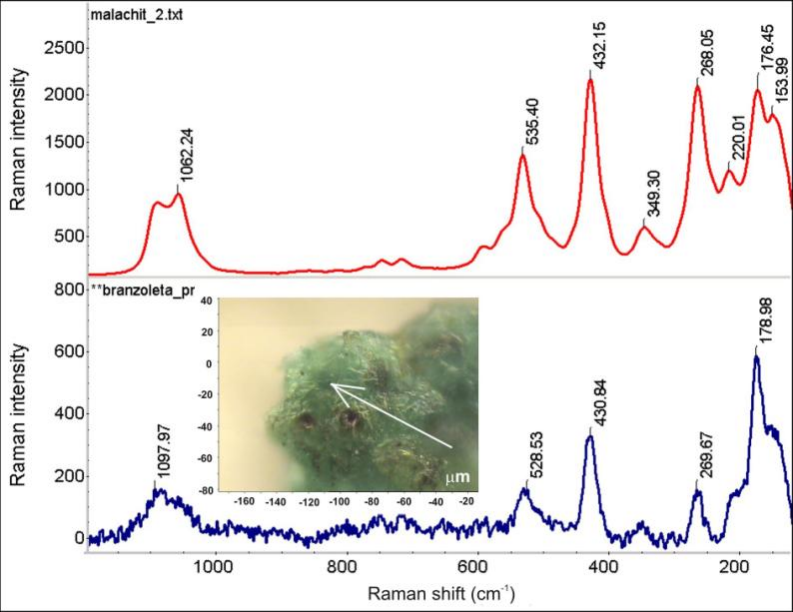
Comparison of the Raman spectrum of malachite Cu_2_(OH)_2_CO_3_ (upper plot) with the Raman spectrum of the green corrosion spot (lower plot) indicated on the small micro-photograph (in the middle)

Figure 23 shows the results of the bracelet sample EDS analysis at the point indicated in the SEM image. The EDS spectrum revealed the presence of products of Al, C, Ca, Cu, S, and Si. The LIBS spectrum, registered during the first accumulation of 20 laser shots (Figure 24) also indicate the presence of Al, Na and Pb. The green spot in the micro-sample was analyzed using a Raman spectrometer (Figure 25). Malachite Cu_2_(OH)_2_CO_3_, copper II hydroxycarbonate is the main spectrum recorded however there is also a thin layer of still unidentified other compounds and quartz present at the external part of the patina.

**Figure 26.**
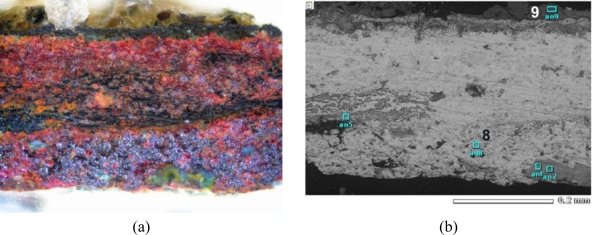
Cross-section images of the micro-sample from bronze fibula: (a) microscope photograph; (b) SEM photograph. Scales are the same.

**Figure 27.**
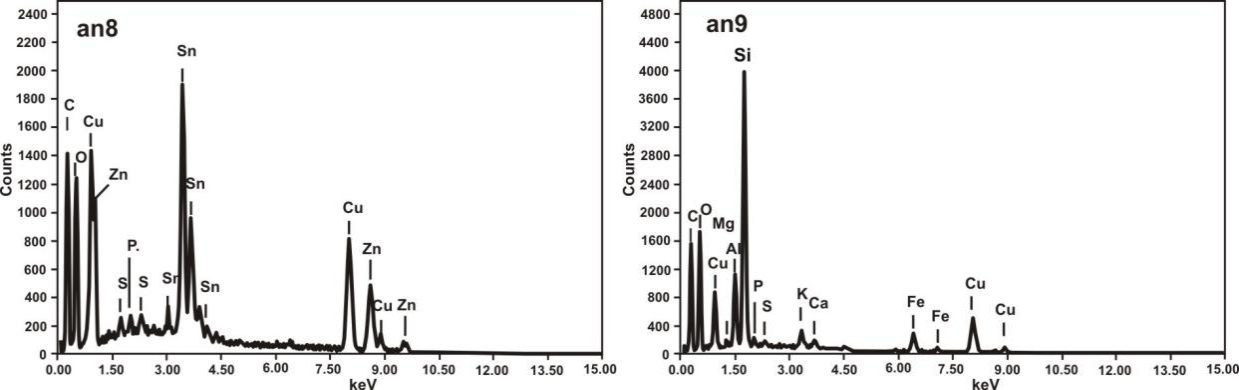
EDS spectrograms of the fibula micro-sample elementary composition at the points indicated in Figure 26(b).

Microscope and SEM images of the microsample taken from the bronze fibula are presented in Figure 26. Points 8 and 9, indicated on the SEM image have been selected to show the results of EDS analysis (Figure 27).

**Figure 28.**
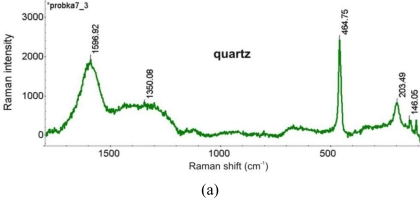

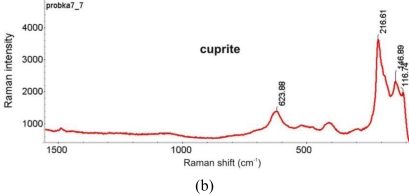
Raman spectra of quartz (a) and cuprite (b), registered on the bronze fibula corrosion layers.

SEM spectrograms, registered near the sample core (point 8), indicate the presence of the main alloy components Cu, Sn and Zn as well as C, O, P and S. Large amount of Si was revealed at the outermost point 9, followed by the presence of C, O, Al, K, Ca and Fe. The Raman spectrum, taken at the area near the SEM sampling point 9 indicated the presence of quartz. Cuprite was identified at the reddish layer shown in Figure 26(a). Both spectra are presented in Figure 28.

#### 3.3.2. Bows from Wilanów Palace Museum in Warsaw

**Figure 29.**
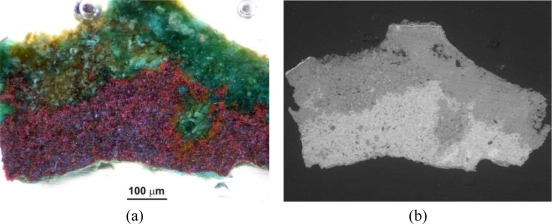
Exemplary images of the bow deposit cross section: (a) microscope photograph; (b) SEM. Scales are almost the same.

**Figure 30.**
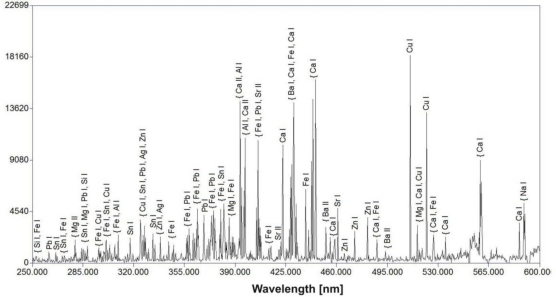
LIBS spectrum of the bow surface deposit.

The two bows present similar degradation patterns and corrosion layers (Figure 29). EDS measurements, together with Cu, have detected many elements such as Si, Al, Fe, Ca, Mg, C, O, P and Pb, thus demonstrating the very strong relationship that exists between soil constituents and degradation products. The complexity of the deposit is demonstrated by the LIBS spectrum of the second bow, shown in Figure 30. Stratigraphic LIBS measurements revealed the surface layer of the environmental elements (e.g., Pb, Ca) with a thickness of around 200 μm (Figure 40). This suggests a long storage period before the performance of the present measurements.

**Figure 32.**
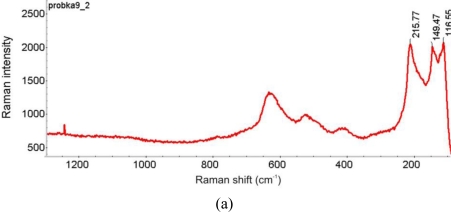

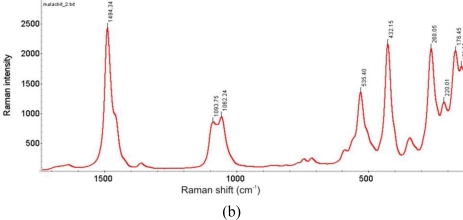
Raman spectra from the reddish layer (a) and green layer (b) of the section shown in Figure 29, indicating the presence of cuprite (a) and malachite (b).

Raman spectra, registered on the bow section presented in Figure 29 were identified as cuprite (reddish layers) and malachite (green layer). Both spectra are presented in Figure 32.

**Figure 33.**
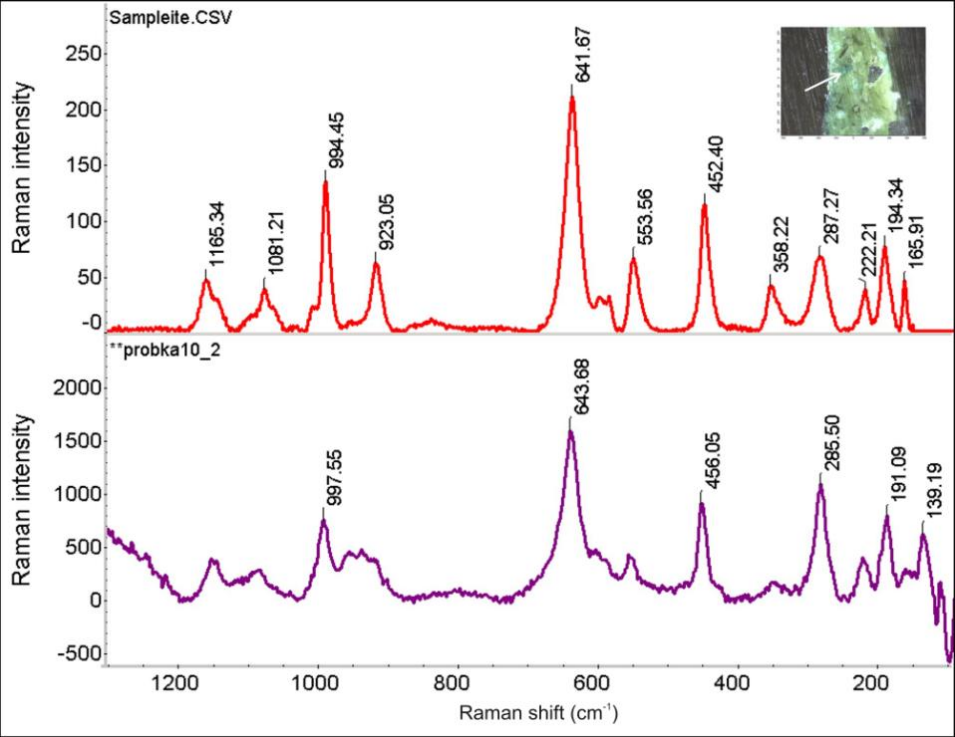
Comparison of the Raman spectrum of sampleite NaCaCu_5_(PO_4_)_4_Cl•5H_2_O (upper plot) with the Raman spectrum from the green corrosion spot (lower plot) indicated on the small micro-photograph (right-top corner).

An interesting copper compound was revealed in the other micro-sample of bow deposit, shown in Figure 33. Sampleite NaCaCu_5_(PO_4_)_4_Cl•5H_2_O has been rarely fully authenticated as a corrosion product of ancient copper alloys [25].

## 4. Conclusions

These investigations have provided evidence of the complex nature of degradation and deterioration of copper-based artefacts in soil and the polluted atmosphere. A detailed review of the corrosion products in ancient copper alloys was beyond the scope of the present paper, however some results showed the importance of further, more complex analysis of selected artworks.

Micro-Raman spectroscopy appears to be a very powerful technique for the identification of local atmospheric corrosion products on copper alloys. However, a lack of a library of reference spectra is also evident, particularly for the analysis of mixed corrosion products. Dozens of Raman spectra, taken during the present work still need further processing.

The presented results show a great usefulness of the LIBS technique for investigating the chemical composition of superficial layers of metallic objects. Apart from common studies of LIBS spectra, the method also allowed for the determination of the stratigraphic composition of layers for all the selected ancient objects. Tests of the putti pair quickly exhibited a different provenance (different alloy used) of the sculpture elements which just goes to show that some elements of the artwork are not original or that some of the conservation processes previously used were not professional. From another point of view, LIBS stratigraphic measurements showed that all the surfaces are covered with an encrustation consisting of, first of all, calcium, than sodium, aluminum and lead. Despite the fact that lead is one of constituents of alloys used in the creation of the artwork, the fact that it is much more highly concentrated in the outer layers confirms the high influence of pollutants originating from the environment on the chemical composition of the surface. The presence of the environmental (Ca, Na, Al, Mg, Pb) encrustations found on copper sheets from the historic palaces in Warsaw and ŁódŸ exhibit the constant destructive influence of the environment on the condition of artworks and monuments.

Comparison of LIBS and Raman spectroscopic results with those found by materials engineering methods show, generally, good qualitative agreement. The results of the first trials demonstrate the effectiveness of an integrated approach employing techniques that give both elemental and molecular information. However, the confirmed potential of materials engineering methods (SEM EDS, XRF) is very useful in the current research-validation of the laser cleaning technique [22,26]. It will allow to study not only changes in the composition of superficial layers, but also morphology of the deposit and the substrate of artwork after gradual laser processing.

## Figures and Tables

**Figuree 13.**
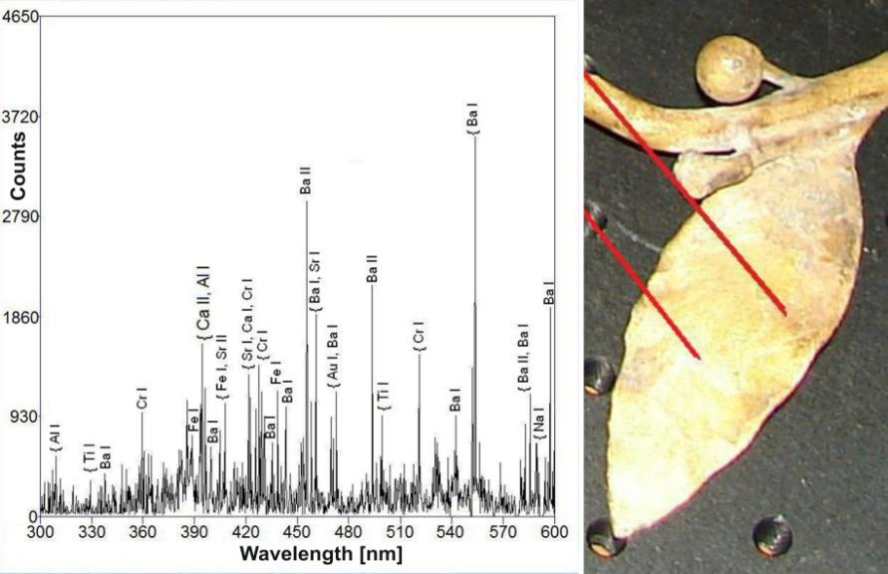
LIBS spectrum of the laurel leaf surface. Right hand side—photograph with the measurement points indicated.

**Figure 31.**
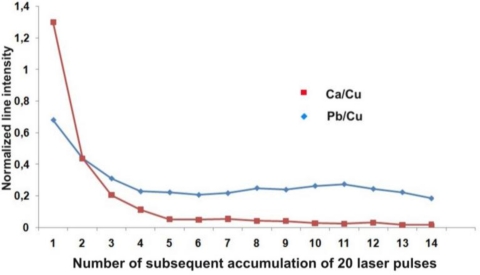
LIBS stratigraphy of Ca and Pb concentration in the deposit.

**Table 1.** Results of EDS point analyses of the surface deposit from the green part of the copper sheet from Wilanów Palace (range of percentage composition determined by measurements from three different points).

**wt.%**	**C**	**O**	**Al**	**Si**	**P**	**S**	**Cl**	**Ca**	**Fe**	**Cu**
lowest	0	25.75	0.35	0.52	0.62	6.37	0.60	0	0	49.68
highest	3.40	37.34	0.48	1.00	0.85	7.27	1.06	0.20	1.02	61.25

**Table 2.** Results of point EDS analysis [wt%] – points from Figure 15.

Point	Cu	O	S	C	Cl	Fe	Ca	Zn
2	40–55	25–35	10		1–5			
3	35–45	25–40	several	*	1–3	1–4	1–1.5	6–11

* – from several to a dozen or so percent depending on the position of the probe.

**Table 3.** Results of LIBS measurements of the original substrate alloy composition of four archaeological objects selected for the studies presented here.

**Object**	**Composition (wt%)**
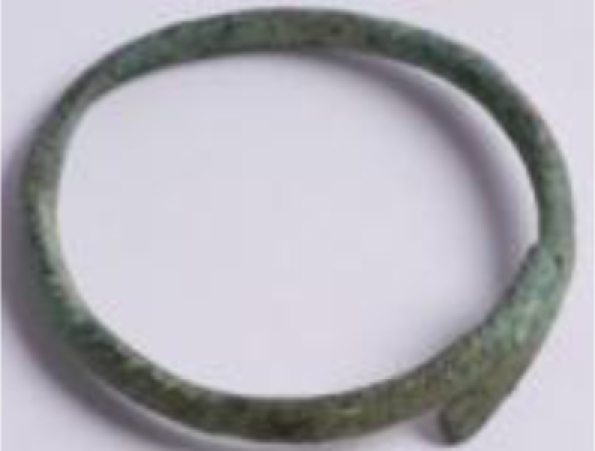	Bracelet made from leaded tin bronze: Cu 82.0; Sn 16.0; Pb 1.5; Fe 0.5
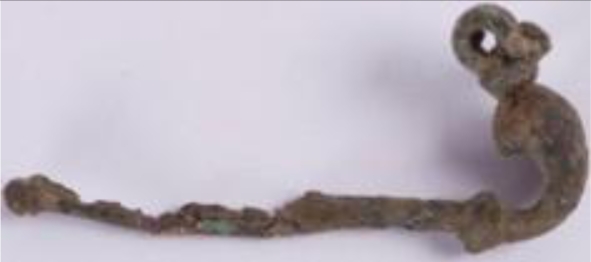	Fibula made from leaded tin bronze: Cu 77.4; Sn 21.5; Pb 1.1
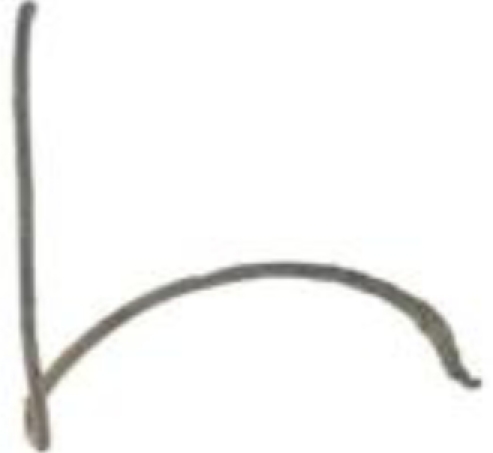	Bow made from leaded tin bronze: Cu 85.0; Sn 11.5; Pb 3.0; Fe 0.5
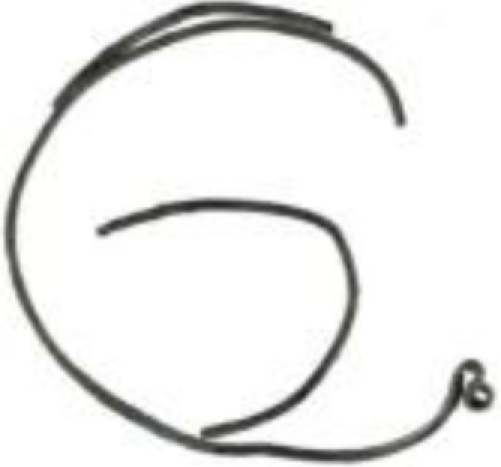	Bow made from red bronze containing copper, tin, zinc and lead: Cu 67.0; Sn 17.5; Zn 12.5; Pb 3.0
